# An epigenetic, transgenerational model of increased mental health disorders in children, adolescents and young adults

**DOI:** 10.1038/s41431-020-00726-4

**Published:** 2020-09-18

**Authors:** Anthony P. Monaco

**Affiliations:** Office of the President, Ballou Hall, Tufts University, Medford, MA 02155 USA

**Keywords:** Genetic variation, ADHD, Addiction, Autism spectrum disorders, Genetic variation

## Abstract

Prevalence rates of mental health disorders in children and adolescents have increased two to threefold from the 1990s to 2016. Some increase in prevalence may stem from changing environmental conditions in the current generation which interact with genes and inherited genetic variants. Current measured genetic variant effects do not explain fully the familial clustering and high heritability estimates in the population. Another model considers environmental conditions shifting in the previous generation, which altered brain circuits epigenetically and were transmitted to offspring via non-DNA-based mechanisms (intergenerational and transgenerational effects). Parental substance use, poor diet and obesity are environmental factors with known epigenetic intergenerational and transgenerational effects, that regulate set points in brain pathways integrating sensory-motor, reward and feeding behaviors. Using summary statistics for eleven neuropsychiatric and three metabolic disorders from 128,989 families, an epigenetic effect explains more of the estimated heritability when a portion of parental environmental effects are transmitted to offspring alongside additive genetic variance.

## Introduction

**Fig. 1:**
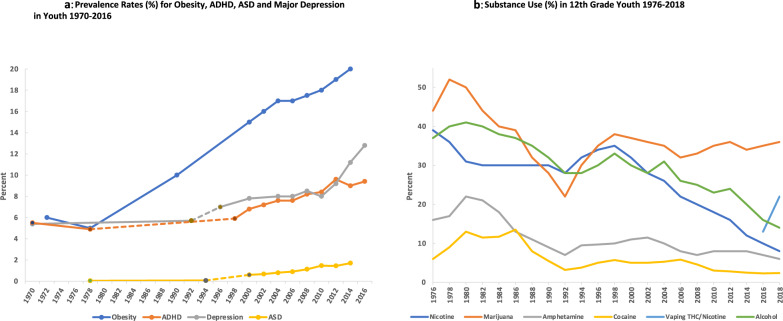
Prevalence Rates for Obesity, ADHD, ASD, Major Depression and Substance Use in Youth. **a** Prevalence Rates for Obesity, ADHD, ASD and Major Depression in Youth 1970–2016. Obesity rates from NCHS, National Health Examination Survey III (ages 12–17) and National Health and Nutrition Examination Surveys (NHANES) I-III, and NHANES 1999–2000, 2001–2002, 2003–2004, 2005–2006, 2007–20008, 2009–2010, 2011–2012, and 2013–2014 (https://www.niddk.nih.gov/health-information/health-statistics/overweight-obesity). Autism spectrum disorder (ASD) rates for 1970s-80s and 1980s-1990s [18]. ASD rates for 2000–2014 were from CDC (https://www.cdc.gov/ncbddd/autism/data.html). The rates for Attention Deficit Hyperactivity Disorder (ADHD) from 1998–2016 were from CDC (https://www.cdc.gov/ncbddd/adhd/timeline.html) National Health Interview Survey (NHIS) annual report 3–17 years of age. The rates for ADHD for 1972 and 1978 were taken from school community teacher surveys [16, 17]. The rates for past-year major depression episode (MDE) DSM-IV criteria are from the 12–17 age group as analyzed by Weinberger et al., 1999 with data from National Survey on Drug Use and Health (NSDUH) [6]. Depression rates for 1970 and 1992 [1]. **b** Substance Use in 12th Grade Youth 1976–2018. Percentage substance use for 12th graders for nicotine by smoking (30-day past use), cocaine (including crack, annual past use) and amphetamine (including Ritalin and Adderall, annual past use), marijuana (annual past use), alcohol bingeing (% who had 5+ drinks in a row at least once in the past 2 weeks) and vaping THC and nicotine (30-day past use) [58]. For cocaine, amphetamine and marijuana, 30-day past use data were only available from 1991 onwards [58].

There has been a two to three-fold rise in prevalence rates for several mental health disorders (MHD) in U.S. children and adolescents over the past twenty years (Fig. 1a, [2000–2017 contiguous data from the Centers for Disease Control, National Health and Nutrition Examination Surveys (NHANES) and National Survey on Drug Use and Health; pre-2000 data points referenced] [1–6]. There has also been a markedly increased rate of suicide in the U.S. since 2008 among the 10–24 year olds, and growth in suicidal ideation or attempts, self-harming behavior, and onset of major mood disorders with acute presentation [7–9].

Proposed contributing factors to increased rates of MHD in children and young adults include adverse childhood experiences, trauma, identity discrimination, financial burden, social media/screen time, substance use, academic/career stress, and other environmental factors and their interaction with individual genetic susceptibility [9, 10]. Population level studies on early childhood screen time and adolescent use of smartphones and social media, showed only weak or no association with decreased mental well-being or increased MHD [11].

It is challenging to test plausible causal hypotheses for the increased prevalence of MHD. This paper will discuss potential epidemiological, genetic, environmental and epigenetic mechanisms, including parental transmission to offspring of shared and unique environmental effects. Human genetic studies of common, complex diseases such as MHD have implicated thousands of genetic variants by genome-wide association and sequencing analysis. Despite adequately powered clinical cohorts, only small increments in risk are generated by individual genetic variants and the overall polygenic effect explains only a fraction of the familial clustering and heritability estimates in the population [10, 12]. Geneticists have turned to whole genome sequencing (WGS) efforts and other approaches to explain the remaining heritability (Supplementary material) [13]. Unknown rare variants may be contributing to disease risk but initial WGS studies of disease cohorts revealed small numbers of functional rare variants with larger cohorts needed [14].

Intergenerational and transgenerational effects via emerging epigenetic mechanisms could also contribute to heritability estimates given the strong behavioral and metabolic effects in rodent models across multiple generations for environmental exposures to stress, toxicants, substances of misuse and obesogenic diet [13, 15]. Heritability estimates for human neuropsychiatric and metabolic disorders will be modeled for transmission of parental environmental effects to offspring alongside additive genetic effects. Environmental factors such as substance use and misuse, poor diet, and obesity will be implicated, as well as convergent brain mechanisms for long-term epigenetic dysregulation, which in rodent models, are transmitted to offspring through intergenerational and transgenerational mechanisms affecting behavioral and metabolic traits.

## Epidemiology

Epidemiologic data for U.S. children and adolescents were reviewed over the past fifty years to understand when the increased rates of MHD diagnoses began. Trend lines for rates of past-year major depressive episode (12–17 years old), obesity (12–17 years old), and parent survey-reported diagnoses (3–17 years old) of Attention Deficit Hyperactivity Disorder (ADHD) and Autism Spectrum Disorder (ASD) are shown in Fig. 1a. Until the mid-1990s, the rates for major depressive episode in adolescents and ADHD in children and adolescents were 4–6%, with ASD in children and adolescents below 0.5% [16–18]. They all trended upwards by 2000, with depression reaching 13% of adolescents in 2016, and in the 3–17-year-old age group, ADHD was 9.4% and ASD was 1.7%. Anxiety disorders were complex to chart over this time period given changes in diagnostic and subtype criteria. However, rates for anxiety disorders, across all age groups, were relatively stable from 1970–1992 [1] with increases seen across many subtypes from 2000–2016 [19–21].

It is difficult to quantify the extent to which increased rates of MHD in children and adolescents were based on (1) steady rises in participation in mental health services due to decreased stigma, (2) increased awareness and better treatments by clinicians, (3) changes in diagnostic criteria, or (4) an underlying increase in incidence and prevalence. Longitudinal and cross-sectional studies have attempted to discern the differences, but the situation is complex [9, 22, 23]. Although the diagnostic landscape for MHD has changed, the significantly increased rate of acute presentation and suicide in adolescents and young adults over the last 10–15 years in the U.S. should not be affected by the first three factors (unless there is a treatment effect on suicide; not all countries have seen increased suicide rates) [7]. Some portion of these striking increases in MHD diagnoses in the U.S. over twenty years could be due to a true increase in incidence and prevalence rates. Recent age:cohort:period analyses of mood disorder rates and suicide-related outcomes in U.S. adolescents and young adults showed a strong cohort effect for those born in the late 1990s who are college-age today [9].

## Mechanisms for increased mental health disorders in children and adolescents

Possible mechanisms for increased MHD include changing environmental conditions, affecting the fetus in utero (intergenerational maternal effects) or early in post-natal life (intragenerational effects on offspring). In animal paradigms, maternal stress or toxic exposures in utero epigenetically altered the fetal genome to change long-term gene expression thereby influencing health and behavior in the progeny [24].

A second intergenerational epigenetic mechanism is bacterial “microbiome” populations, most notably in the intestinal and urogenital tracts. In humans, an unhealthy maternal microbiome has been shown to increase pre-term births, and intestinal flora have been implicated in neurological and metabolic disorders [25–27]. In mouse models, maternal stress effects on vaginal microbiota influenced the health and behavior of male progeny with gene expression changes in the hypothalamus [28]. Also, a poor maternal diet led to gut microbiome changes in male mouse offspring, inducing social deficits which were restored via reintroduction of a commensal bacterial strain [29].

A third potential mechanism is long-term, epigenetic changes that influence health and behavior across multiple generations (intergenerational and transgenerational effects defined by number of generations affected) [15, 24, 30, 31]. There are many rodent models of stress, rewarding foods and substances of misuse which can alter synaptic plasticity in specific brain circuits and influence the health and behavior of the next generation progeny (F1 for father exposed) and remarkably, the third-generation (F2) [15, 32–38]. In mice, the transmission of the negative consequences of a poor diet in males were due to changes in the content sperm small non-coding RNAs (ncRNAs) including microRNA (miRNA) and transfer RNA fragments [39]. Recent evidence using a paternal transgenerational stress model in mice, showed that small ncRNAs, specifically miRNA ratios in sperm, provided the epigenetic information to influence progeny behavior [40]. Changes in sperm miRNAs found in mouse stress models were correlated with early life stress in humans [41].

miRNAs are small single-stranded regulatory ncRNAs (21–25 nucleotides) involved in controlling protein levels by binding to the 3’-untranslated region of messenger RNAs and enhancing their decay before translation can initiate and also by direct ribosomal translation inhibition [42]. They are found in multiple cellular compartments and released upon stress via exosomes (along with other ncRNAs and cell-specific protein and lipid cargos) thereby influencing cell–cell communication [43]. miRNAs are stable over time and along with other ncRNAs, are candidates for enduring regulators of developmental, regenerative, metabolic, brain, and infectious states in response to changing environmental conditions. miRNAs are enriched in the epididymis and secreted by luminal principal cells via epididymosomes with other ncRNAs and protein cargoes, influencing the luminal environment in anatomically distinct compartments. Epididymosome cargoes together with other epigenetic mechanisms in developing spermatozoa, could influence genome regulation in the zygote and developing fetus [44, 45]. There is also evidence for exosome vesicles secreted by the oviduct and embryo, opening up novel lines of communication and regulation in the gametes–embryo–oviduct environment [46].

Current studies are aimed at understanding mechanisms in vertebrates by which environmental changes are integrated over time to generate deviations in gamete epigenetic marks. A central question is whether the nervous system provides the sensory integration to signal changes to gametes via neuronal, exosome or hormonal pathways, or the cellular environment of gamete development is able to sense and integrate environmental factors directly. In the worm *C. elegans*, a detailed RNA-based mechanism was shown for neuronal activity directly effecting germline ncRNA content and the behavior of the next generation [47]. Recently, direct evidence for transmission of miRNAs was demonstrated from male mouse brains to the germline and one-third of F1 embryos [48]. In addition to ncRNA mechanisms, there are examples of stable transmission of chromatin condensation, histone modifications, and DNA-methylation which are transmitted to offspring via gametes, which have been reviewed extensively [24, 30, 35].

In humans, there is evidence for transgenerational effects of trauma, adverse childhood experiences and Post-Traumatic Stress Disorder [49, 50]. Children of Holocaust survivors, for example, experienced epigenetic changes within the hypothalamic–pituitary–adrenal axis [51]. The phenomenon of transgenerational inheritance has also been attributed to famine undernutrition in the Netherlands in WWII. There were significant increases in adiposity and other health issues not only in the second generation but in grandchildren of those affected by the famine [52]. Swedish studies also provided evidence for rapid changes in food supply having a possible transgenerational effect from maternal grandmothers through their sons to poor cardiovascular outcomes in maternal granddaughters [53]. Analysis of Swedish adoption registries revealed a biological parental environment effect transmitted to offspring with alcohol use disorder alongside genetic and parental rearing environment effects [54]. Using the ALSPAC cohort, paternal early age cigarette smoking was shown to significantly increased body mass in adult children, which was stronger for male offspring [31]. There is evidence for transgenerational effects in domesticated farm animals and birds from parental exposures to environmental toxicants, nutrients and infectious agents [55, 56].

## Potential environmental factors for intergenerational and transgenerational effects

Substance use and misuse (including alcohol and smoking), poor diet, and obesity are three environmental conditions with potential long-term epigenetic and transgenerational effects which could have influenced rates of MHD in the current generation. These three factors were already implicated as major environmental contributors to decreased life expectancy in the U.S. from 2015 to 2017. The highest specific contributions were from drug overdose, suicide, and alcohol- and obesity-driven organ system disease, with increases dating back to the 1990s [8, 57]. The strongest environmental contributors to decreased morbidity and mortality in the U.S. population may also have contributed an intergenerational or transgenerational effect to health outcomes in the next generation.

Nicotine, caffeine, stimulants, and marijuana were all used frequently in the population during the 1970–80s (Fig. 1b) [58, 59]. Nicotine use declined nearly fourfold between the late 1970s, when 30–40% of youth and adult populations reported use, to <10% in 2018 [58, 59]. In the 1970–80s there was a very large increase in high school students and young adults in the U.S. using marijuana, stimulants and binge-drinking alcohol (Fig. 1b) [58, 59]. Cocaine, amphetamine(s), and methylphenidate were taken recreationally or for performance-enhancement, in a significant portion of the population during the 10-year period from 1976 to 1986. Caffeine intake was high throughout the past 50 years but consumption of high sugar caffeinated soft drinks, particularly in children, increased dramatically in the 1970–80s to peak at the turn of the century [60, 61]. Heroin was used less frequently during the 1970–80s, but significant increases in opioid addiction started after the introduction of long-acting prescription pain relievers in the 1990s with many users switching to heroin following prescription tightening habits, in combination with other substances like fentanyl, resulting in higher drug overdose deaths since 2000 [8, 58, 59, 62].

Heritability estimates for substance use disorders (SUD) have shown specific and pleiotropic genetic effects for all substances with cocaine and marijuana the strongest, modest levels for nicotine and alcohol, and lowest levels for caffeine [63]. Risk for SUD comprised contributions from both genetic variants (nicotinic acetylcholine receptor variants in SUD) and environmental influences (parental control, permissive environment for access). The environmental effects changed from childhood to adolescence, and indirect effects of gene-environment correlations are plausible where disorder-specific behaviors increased the environmental exposures, as seen in ADHD and SUD [63].

In addition to increased substance misuse, a second major societal trend involved dramatic changes to the U.S. food supply and lifestyle after the 1960s [60]. This comprised major rises in availability and consumption of ultraprocessed foods (rich in refined starch, sugar and additives), high sugar caffeinated soft drinks, alcohol, and other factors such as continued physical inactivity and sedentary lifestyle. In the 1980s, childhood obesity in the U.S. was 5%, and rose rapidly to 20% today (NHANES, 3–17-year olds, Fig. 1a). Poor diet and obesity should be considered as correlated, but separable environmental conditions with both anatomical and temporal differences in potential brain effects. The increase in childhood and adolescent obesity (NHANES, Fig. 1a) and peak of substance use and misuse (Fig. 1b), occurred 15–20 years before the steady increases in MHD, providing adequate time for a transgenerational phenomenon to have occurred affecting this generation.

Changes in diet and accompanying obesity, in addition to high prevalence of substance misuse, may have had intergenerational and transgenerational effects, which are now manifesting themselves as new set points in brain circuits for highly regulated sensory, motor, reward and feeding systems. These new set points and relative sensitization of specific brain circuits could contribute epigenetically to increased risk for MHD. In rodent models of stress, obesogenic diet, and substance misuse (methamphetamine, cocaine, and opioids), miRNA levels have been shown to be dynamically regulated in the ventral basal ganglia, and in the methamphetamine model, corresponded to specific shared miRNA levels in circulating serum exosomes [64–68].

Given their rewarding and possibly addictive nature, ultraprocessed, high glycemic index foods may have contributed to dysregulation of reward pathways in the basal ganglia to prefrontal cortex and limbic system [69]. There is convincing evidence that palatable foods, with high glycemic index, stimulate the same reward pathways in the basal ganglia as all of the substances of misuse in both humans and animal models [69, 70]. Substances of misuse, ultraprocessed high glycemic index foods and high sugar caffeinated drinks could have acted cooperatively to sensitize the cortico–striatal–limbic and cortico–striatal–thalamic pathways to further rounds of these environmental conditions, which are transmitted as new set points to the next generation.

## Test of the transgenerational model

From genetic studies of common diseases with large cohort sizes, the genetic variance effect size of individual genome-wide significant variants is small and the overall polygenic variation (*G*_*A*_) is also relatively small (0.05–0.3) [10, 71]. When the effect size is measured across multiple large single-nucleotide polymorphism (SNP) genotyping studies for five common MHD, estimates of $G_{SNP}$ for schizophrenia (0.23), bipolar disorder (0.25), major depressive disorder (0.21), ASD (0.17), and ADHD (0.28) plus known CNV contributions, showed that only 36–48% of the heritability is explained by $G_A = G_{SNP} + {\mathrm{CNVs}}$ (Table 1) [10, 14, 72–74].

**Table 1:** Residual heritability for five neuropsychiatric disorders.

Disorder	ASD	ADHD	Bipolar disorder	Depression	Schizophrenia
$H^2$	0.924	0.763	0.676	0.579	0.562
$G_A$ measured ($G_{SNP}$ + CNVs)	0.37 (0.17 + 0.2) 40%	0.30 (0.28 + 0.02) 39.3%	0.25 37%	0.21 36%	0.27 (0.23 + 0.04) 48%
Residual *H*^2^	0.554 60%	0.463 60.7%	0.426 63%	0.369 63.7%	0.292 52%
Estimated *G* effect [$G_A(0.5\,E_P) + 0.5\,E_P$]	0.924	0.568 (0.468 + 0.1) 74.4%	0.468 (0.333 + 0.135) 69.2%	0.373 (0.192 +0.181) 64.2%	0.435 (0.308 +0.127) 77.4%
Residual $H^2$	0	0.195 25.6%	0.208 30.8%	0.207 35.8%	0.127 22.6%

Heritability $H^2$ [72], measured $G_A = G_{SNP} + {\mathrm{CNVs}}$ [10, 14, 73, 74], Residual $H^2$, Estimated $G\left( {\sigma _G^2} \right) = \left( {G_A + 0.5E_P} \right)$ effects (this paper) and Residual $H^2$ for five major psychiatric disorders. All % are for portion of $H^2$.

In Supplementary material, the possible reasons for the remaining heritability are discussed including additional rare variants and possible intergenerational and transgenerational effects. If a portion of the parental shared environmental variance is contributing to a transgenerational effect, then a first approximation for broad-sense heritability $\left( {H^2} \right)$ can be estimated from the first-order direct genetic and environmental variances only:

1 $$H^2 \cong \frac{{G_A + xE_P}}{{G_A + E_P + E_O}} \cong \frac{{G_A + xE_P}}{{G_A + E}},$$

where $E = xE_P + \left( {1 - x} \right)E_P + E_O = E_P + E_F + E_S + E_U,\,0 < x < 1$ and $E_O = {\mathrm{offspring}}\,{\mathrm{environmental}},\,{\mathrm{E}}_P = {\mathrm{shared}}\,{\mathrm{parental}},{\mathrm{E}}_F = {\mathrm{shared}}\,{\mathrm{familial}}$, ${\mathrm{E}}_S = {\mathrm{shared}}\,{\mathrm{sibling}},\,{\mathrm{E}}_U = {\mathrm{unique}}\,{\mathrm{environmental}}\,{\mathrm{variances}}$, $0 < x < 1$ (Supplementary material).

Empirical data from 40 million U.S. insurance claims for 149 common diseases was used to estimate liability-scale heritability and environmental variances in 481,657 unique individuals from 128,989 families where both parents and children were covered for at least 6 years [72]. To test the transgenerational model, eleven neuropsychiatric and three metabolic disorders where chosen from this large-scale study with high heritability estimates (avg. 0.60, range 0.422–0.924), comparable with most meta-analyses from either family or twin studies, with some variability. For these fourteen disorders, the average shared parental environmental effect ($E_P$) is high (0.27), 63% of the total environmental variance (*E*), with autism being an outlier with no shared parental environmental effect (Supplementary material). In addition, for 21 neuropsychiatric disorder-pairs, the environmental correlations ($\overline {r_e} = 0.39$) were nearly as strong as the genetic correlations ($\overline {r_g} = 0.5$) with clear groupings of MHD reflected in the resulting nosology classification (Supplementary material) [72].

To test the transgenerational model, where $E_P$ would contribute some fraction of its variance effect to the total genetic variance $G$ along with additive genetic variance $G_A$, summary statistics for heritability ($H^2$), environmental variance effects from couples (*E*_*P*_), siblings ($E_S$) and unique environmental effects ($E_U$) were selected from Wang et al, (2017) for eleven neuropsychiatric and three metabolic disorders [72]. For each disorder $G$ was calculated (Supplementary material) under four models of $xE_P$ contributing to the numerator in equation number 4 with $x = 0.25,\,0.5,\,0.6\,{\mathrm{or}}\,0.667$.

**Fig. 2:**
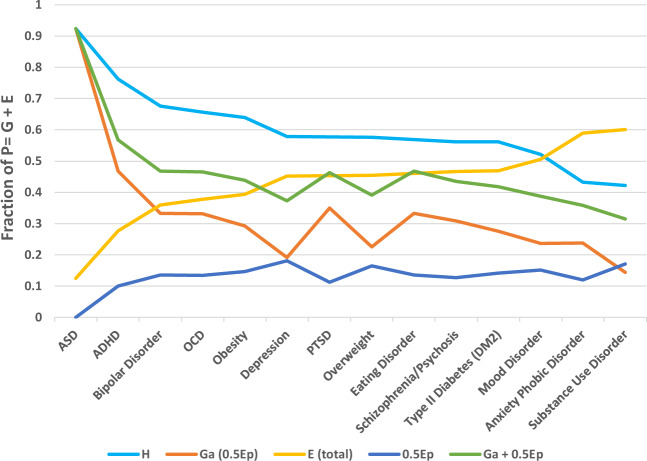
Graph of 0.5*Ep* transmission effect for 11 neuropsychiatric disorders and 3 metabolic disorders by decreasing heritability estimate. Shows the effects of adding 0.5 *E*_*P*_ into the total genetic variance *G* and calculated additive genetic variance (*G*_*A*_) using estimates for heritability (*H*^2^), shared parental environmental variance (0.5 *E*_*P*_) and total environmental variance (*E*) by decreasing order of heritability for 11 neuropsychiatric disorders and 3 metabolic disorders [72].

A transgenerational model of $E_P$ contributing some fraction of its effect to total genetic variance $G$, decreases the contribution of estimated additive genetic variance $G_A$ to heritability. The 0.5 $E_P$ model showed $G_A \ge xE_P$ in its contribution to $G$ for most disorders, and estimated $G_A$ values aligned with current $G_{SNP} + {\mathrm{CNV}}$ effects from genetic association and sequencing data with room for additional genetic effects from WGS discovery of rare variants. Figure 2 presents the relationship of $G_A$ and $0.5E_P$ in the transgenerational model by disorder heritability from highest to lowest. The additive effects of $G_A$ and $xE_P$ to $G$ do significantly close the gap in the remaining heritability, but do not explain it fully.

Table 1 shows estimated liability-scale heritability estimates for five neuropsychiatric disorders with $H^2$ above 0.56 [72] and remaining heritability after accounting for measured $G_A = G_{SNP}$ from the largest meta-analysis [10] and percentage of CNVs contributing to the disorder [14, 73, 74]. It also shows $G\sim (G_A + 0.5E_P)$ calculated using summary statistics for $H^2$, $E$, and $E_P$ [72] in heritability equation number (1). This model shows a good fit with measured genetic effects of $G_A$ from $G_{SNP}$, rare variants and CNVs. Excluding autism as an outlier given the low contribution of total environmental variance $(E)$ (and specifically $E_P$), the other MHD show about 0.127–0.208 (22–36%) of the heritability not accounted for by this hybrid model. In the traditional model, measured $G_A$ explains 36–48% of $H^2$, where the proposed transgenerational hybrid model ($G_A$ + 0.5$E_P$), explains 64–77% of $H^2$. The remainder may be explained by variation in phenotypic trait diagnoses, assortative mating, unobserved rare variants and second-order effects such as genetic epistasis and gene–environment interactions and covariances.

Analysis of variance of large empirical family datasets such as Wang et al. [72] under a genetic and transgenerational hybrid model would predict a better fit to the data if the shared parental environmental effects are biologically contributing to heritability estimates. It would also estimate more accurately $G_A$, $E_P\,{\mathrm{and}}\,E_U$ variance effects on broad-sense heritability than these arithmetic models might predict being constrained by $H^2$ calculated under the traditional narrow-sense heritability model in Wang et al. [72].

## Conclusion

**Fig. 3:**
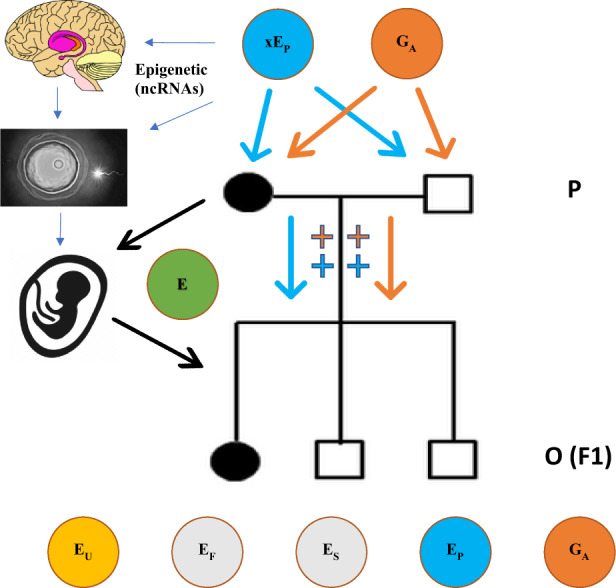
Schematic diagram of direct genetic and environmental variance transmission from parents to offspring and maternal effects in utero of environmental effects. The schematic diagram shows how additive genetic variance (*G*_*A*_, orange) and shared parental environmental variance (x*E*_*P*_, blue) could be transmitted to offspring (O) to influence disorder liability alongside additive genetic and environmental effects in offspring $\left( {P = G_A + E_P + E_S + E_F + E_U} \right)$. Filled circles are affected females (mother and daughter) in this hypothetical example pedigree with empty squares being unaffected males (father and two sons). Environmental influences on the developing fetus (green) would include continuation of any prenatal maternal environmental exposures and any new shared maternal or familial environmental variance.

The effects of dietary and life-style changes in the 1970–80s initiated an obesity epidemic still being felt today. Daily consumption of poor diet, and high prevalence of nicotine, caffeine, marijuana, alcohol, and stimulant drugs in the 1970–80s hypothetically produced long-term changes to the reward and fronto-striatal pathways in the brain in a significant portion of the population. The chronic effects of basal ganglia dysregulation altered sensitization for reward and stress in the brain, with concomitant changes in gamete epigenetic marks, influencing prevalence rates for MHD in youth from the late 1990s until the present day. A intergenerational and transgenerational model (Fig. 3) for parental environmental effects working through an epigenetic code in gametes (ncRNAs and other mechanisms) in combination with additive genetic variance from DNA variants, could explain some of the remaining heritability from human genetic studies.

If this mechanism is contributing, then the next generation may continue to experience high rates of MHD given the continued rise in youth and adult obesity, and substance use and misuse (especially the recent upsurge of vaping nicotine and THC, Fig. 1b and opioid addiction since 2000) [58]. The model predicts that the brain–gastrointestinal–urogenital axis may be essential to sense shifting environmental conditions, modulate behavior through specific circuits in the brain, and transmit epigenetic signals to the next generation. Recent longitudinal analysis in Denmark of MHD with onset of medical conditions found the strongest correlations (mean hazard risks of 3.62) for urogenital conditions and eating disorders, and strong correlations with circulatory, gastrointestinal and specific neurological disorders such as epilepsy and Parkinson’s [75].

Epigenetic intergenerational and transgenerational changes are reversible, and studies are needed to explore both the effect of prevention of exposures in parents, and interventions in offspring at risk for mental health and correlated health disorders.

## Supplementary information

Supplementary Material
