# Supplementary material for: An epigenetic, transgenerational model of increased mental health disorders in children, adolescents and young adults
Source: Eur J Hum Genet. 2020 Sep 18;29(3):387–95. doi: 10.1038/s41431-020-00726-4 (PMC7940651; doi:10.1038/s41431-020-00726-4)
Supplement: Supplementary file 1 — Supplementary Material [file 41431_2020_726_MOESM1_ESM.pdf]

Supplementary Figure 1 and Table 1

Intergenerational and transgenerational effects contribute to heritability

Broad-sense heritability (H^2) for any disorder is defined as the contribution of genetic variance ($G = \sigma_G^2$) to total phenotypic variance ($P = \sigma_P^2$) given a constant environment (1):

$$H^2 = \frac{G}{P} = \frac{\sigma_G^2}{\sigma_P^2} \qquad [1]$$

Total phenotypic variance is derived from genetic variance (σ_G^2), total environmental variance (σ_E^2) and gene by environment interactions (σ_{G*E}^2):

$\sigma_P^2 = \sigma_G^2 + \sigma_E^2 + \sigma_{G*E}^2$. Genetic variance ($\sigma_G^2$) is usually defined by the narrow-sense heritability (h^2), defined by additive genetic variance (σ_A^2) of all individual genetic loci ($\sigma_{A_i}^2$) associated with the disorder assuming no dominance effects ($\sigma_D^2 = 0$) (2). These include the full frequency range from rare to common variants as well as copy number variants (CNVs): $G_A = \sigma_A^2 = \left(\sigma_{A\,1}^2 + \sigma_{A\,2}^2 + \sigma_{A\,3}^2 \ldots \sigma_{A\,n}^2\right) = \sum_{i=1}^{n} 2p_i(1-p_i)\alpha_i^2$ where $2p_i(1-p_i)$ is the heterozygosity of the ith locus with allele frequency p_i, and average effect size α_i in a two-allele system (3). From genetic studies of common diseases with large cohort sizes, the genetic variance effect size of individual genome-wide significant variants ($\sigma_{A_i}^2$) is small and the overall polygenic variation G_A (σ_A^2) is also relatively small (0.05 - 0.3) (4, 5). Possible sources for the remaining heritability include:

1. Nonrandom mating: Occurs when mating pairs are more similar for phenotypic traits than expected at random in the population. This can be due to assortative mating, marital interaction and social homogamy. In heritable MHD disorders, increased concordance of traits between mates through phenotypic assortative mating would add confounding additive genetic variance to heritability due to 'between' parent genotype covariances ($2\sigma_{G,G}$)

resulting in offspring with increased genetic variance, more extreme phenotypes and familial clustering of disorders (6, 7). Assortative mating for traits with lower heritability (social homogamy), such as religion and social class, would increase trait similarity in the next generation while maintaining population subgroups. Phenotypic assortative mating occurring within families showing strong familial clustering of MHD, high heritability and low population prevalence such as autism spectrum disorder (ASD), would increase population prevalence in the next generation, but have only a modest effect on heritability (7). At equilibrium over several generations, it would not explain a population level increase in MHD unless changing environmental conditions influenced mating concordance.

2. ‘Genetic nurturing’: In addition to effects of transmitted parental alleles to offspring contributing to additive genetic variance, there is a significant effect of non-transmitted alleles on phenotypic variance termed ‘genetic nurturing’ (8). More of the phenotypic variance is explained by alleles both transmitted and non-transmitted from parents to offspring. In addition, modelling showed even stronger amplification from genetic nurturing alleles to induced phenotype correlations between parents and offspring, and between sibling-pairs. For example, covariance between transmitted and non-transmitted genotypes of the mother and the environment in utero (intergenerational) or within the family rearing environment (intragenerational) can influence phenotype outcomes (9).
3. Additive genetic variance and rare variants: Whole Genome Sequencing (WGS) and exome sequencing has identified rare variants in common diseases, each of which may be contributing with stronger phenotypic effect size to the narrow-sense heritability h^2 (10). Large numbers of rare variants are needed (requiring linearly scaled sample sizes), each contributing in 0.05 – 0.1% of the population (11). Recent analysis of WGS and exome

sequencing for highly polygenic traits (height, BMI, metabolic enzyme and blood cell data) showed that rare variants, especially those not in linkage disequilibrium with genotyped or imputed common variants, may contribute significantly to additive genetic variance and common trait associations (12, 13). This strategy may not be successful in clinically diagnosed binary or latent class traits with lower heritability, where rare variants and CNVs have yet to contribute significantly to G_A.

4. Non-additive genetic variance:
    a. Genetic epistasis: Non-additive genetic variance from second-order, gene by gene interactions ($G * G = \sigma^2_{G*G}$) where a genetic variant influences the phenotype by interacting with a genetic variant at another locus. The multiple of the individual variant frequencies makes each interaction a rarer event with many genetic interaction-pairs needed for the remaining heritability with adequately powered sample sizes (3, 14).
    b. Dominance effects: Non-additive genetic variance can contribute to phenotypic variance due to interaction of alleles at the same locus (σ^2_D). Contributions to phenotypic variation from dominance effects were thought to be small in animals but recent work in yeast illustrates that one-third of phenotypic variance was due to non-additive genetic effects with important contributions from dominance and partial dominance at some loci under specific environmental conditions (15). Interactions between alleles in loci specific haplotypes in animals and humans could be modulated by particular environmental factors to contribute non-additively to genetic variance.

If epistasis, dominance and genotype covariances are included, then genetic variance is: $\sigma_G^2 = \sigma_A^2 + \sigma_D^2 + \sigma_{G*G}^2 + 2\sigma_{G,G}$ where $2\sigma_{G,G}$ is phenotypic assortative mating of parental genotypes contributing to genetic and phenotypic variance.

c. Gene - environment interactions and covariances: Sensitivity to environmental conditions in both parents and offspring can change gene expression over long time periods, alter regulatory functions and contribute with different temporal trajectories to the phenotype. Environmental exposure substructure in the population (e.g. substance misuse) could interact with inherited genetic variants (e.g. nicotinic acetylcholine receptor) modifying the effects of certain genes to influence phenotypic variation in offspring (1, 16). Parental environmental variance shared 'between' families in a population could add to genetic variance and heritability if the environmental condition acts a linear modulator of additive genetic variance, with genetic influences increasing at extreme values of the moderator (9). 'Within' families, parental environmental variance can also contribute non-additively through genotype - environment covariance ($\sigma_{G,E}$) where trait-influencing genotypes are more likely to be present in certain environments.

$$H^2 = \frac{\sigma_A^2 + \sigma_D^2 + \sigma_{G*G}^2 + 2\sigma_{G,G} + \sigma_{G*E_P}^2 + 2\sigma_{G,E_P}}{\sigma_A^2 + \sigma_D^2 + \sigma_{G*G}^2 + 2\sigma_{G,G} + \sigma_E^2 + \sigma_{G*E}^2 + 2\sigma_{G,E}} \qquad [2]$$

5. Direct intergenerational and transgenerational effects. In rodent models, specific parental environmental conditions can affect the health and behavior of F1 progeny, and even F2 and F3 progeny, through an intergenerational or transgenerational mechanism utilizing small non-coding RNAs (ncRNAs) and other means as an epigenetic code. Some portion of shared parental environmental variance (E_P) could influence total phenotypic variance in the offspring (F1) due to direct parental environmental effects ($E_P = \sigma_{E_P}^2$) on ncRNA levels (and

other mechanisms) transmitted to F1 and possibly F2 progeny without reference to genetic variants. The effect size would not be dependent on genetic variant frequencies, only the environmental exposure threshold in the population and could therefore make a significant contribution to total phenotypic variance.

Not all shared parental environmental variance E_P ($\sigma^2_{E_P}$) would be contributing via a transgenerational effect to heritability estimates. A large portion of E_P (parental rearing or nurturing environment) is contributing to trait liability in offspring either directly, by gene-environment interactions or covariances. For example, substance misuse by parents could lead to a direct transgenerational effect, an increased parental exposure driven by genotype and a permissive rearing environment leading to increased substance use by their children, in addition to additive genetic susceptibility and unique offspring exposure. So only a portion of the parental environmental variance ($xE_P = x\sigma^2_{E_P}$) should be considered as contributing to heritability.

The parental environmental transmission effects (direct, interactions and covariances) would add to the total genotypic effect from parents and contribute to total phenotypic variance in the offspring (O):

$$H^2 = \frac{(\sigma^2_A + \sigma^2_D + \sigma^2_{G*G} + 2\sigma_{G,G}) + (x\sigma^2_{E_P} + \sigma^2_{G*xE_P} + 2\sigma_{G,xE_P})}{(\sigma^2_A + \sigma^2_D + \sigma^2_{G*G} + 2\sigma_{G,G}) + (\sigma^2_{E_P} + \sigma^2_{G*E_P} + 2\sigma_{G,E_P}) + (\sigma^2_{E_O} + \sigma^2_{G*E_O} + 2\sigma_{G,E_O})} \quad [3]$$

where $\sigma^2_E = x\sigma^2_{E_P} + (1-x)\sigma^2_{E_P} + \sigma^2_{E_O} = \sigma^2_{E_P} + \sigma^2_{E_F} + \sigma^2_{E_S} + \sigma^2_{E_U}, 0 < x < 1$ and $\sigma^2_{E_O} =$ offspring environmental, $\sigma^2_{E_P} =$ shared parental, $\sigma^2_{E_F} =$ shared familial, $\sigma^2_{E_S} =$ shared sibling, $\sigma^2_{E_U} =$ unique environmental variances, $0 < x < 1$.

Modeling of empirical data has shown that dominance genetic effects (σ^2_D) and epistatic interactions (σ^2_{G*G}) do not contribute significantly to heritability, or for epistasis, we need power

function-scaled sample sizes to detect them (2, 5, 11, 14, 17). In yeast, exhaustive searches for genetic interaction effects across twelve traits found 9% of the phenotypic variance was due to pair-wise gene-gene epistasis (18). Therefore, current measurable genetic effects are almost entirely through additive genetic variance G_A (σ_A^2). It is difficult to estimate effect sizes for genotypic covariances (assortative mating, other genotype correlations), and gene - environment interactions and covariances due to many parameters being unknown or unobserved. For a first approximation, H^2 can be estimated from only the first-order direct genetic and environmental variances:

$$H^2 \cong \frac{\sigma_A^2 + x\sigma_{E_P}^2}{\sigma_A^2 + \sigma_{E_P}^2 + \sigma_{E_O}^2} \cong \frac{\sigma_A^2 + x\sigma_{E_P}^2}{\sigma_A^2 + \sigma_E^2} \cong \frac{G_A + xE_P}{G_A + E} \qquad [4]$$

Environmental effects in ASD and ADHD. Both disorders have extremely high heritability estimates, yet very little or no shared parental environmental variance effects ($\sigma_{E_P}^2$). Shared parental environmental variance ($\sigma_{E_P}^2$) between families could be interpreted as additive analogous to additive genetic variance G_A (σ_A^2). But shared environmental variance may not include a strong transgenerational effect from a unique parental environmental variance effect ($\sigma_{E_U}^2$), which may act dominantly. ASD and ADHD could exhibit a dominance effect of unique environmental exposures ($y\sigma_{E_U}^2$) in one or both of the parents, which can be transmitted epigenetically as increased risk in offspring alongside relatively weak additive shared environmental and very strong additive genetic variance from parents (19). As noted below, ADHD exhibited weaker genetic and environmental correlations with other psychiatric disorders that exhibited very high pairwise genetic and environmental correlation coefficients with each

other (bipolar, mood, schizophrenia, depression and anxiety/phobic disorders (20). The equation for heritability would then become:

$$H^2 \cong \frac{\sigma_A^2 + x\sigma_{E_P}^2 + y\sigma_{E_U}^2}{\sigma_A^2 + \sigma_E^2} \text{ and } \sigma_E^2 = x\sigma_{E_P}^2 + (1-x)\sigma_{E_P}^2 + \sigma_{E_F}^2 + \sigma_{E_S}^2 + y\sigma_{E_U}^2 + (1-y)\sigma_{E_U}^2, 0 < \frac{x}{y} < 1 \quad [5]$$

Genetic, environmental and phenotypic correlations between seven neuropsychiatric disorders from Wang et al (2017). Correlation coefficients for genetic (orange), environmental (blue) and phenotypic variance (gray) are compared between seven neuropsychiatric disorders (Supplementary Figure 1). For 21 neuropsychiatric disorder-pairs, the environmental correlations ($\overline{r_e} = 0.39$) were nearly as strong as the genetic correlations ($\overline{r_g} = 0.5$)(20). There appear to be three potential groupings of disorder correlations.

1. Bipolar disorder, mood disorder, schizophrenia, anxiety/phobic disorders and depression have high H^2 (avg. 0.55), high E_P (avg. 0.29), and high $\overline{r_g}$ (avg. 0.62) and $\overline{r_e}$ (avg. 0.47). These disorders may all share strong pleiotropic additive genetic variance and an environmental variance transmission effect from parents to offspring derived from high shared E_P and the very high $\overline{r_e}$ between them.
2. The second group is clustered around ADHD (very high H^2= 0.76) but lower $\overline{r_g}$ (0.35) and $\overline{r_e}$ (0.26) with all other neuropsychiatric disorder pairs. Given ADHD is frequently co-morbid for anxiety/phobic disorder, depression and SUD, it could be influenced more by unique parental environmental factors (see above), which increase risk for early onset ADHD and later onset co-morbid MHD, however, differently from the first group of disorders (1) given its lower $\overline{r_e}$ and $\overline{r_g}$with them.
3. The third group is SUD with strong genetic and environmental correlations with all other MHD ($\overline{r_g}$ = 0.44; $\overline{r_e}$ = 0.38), and higher than those for ADHD-MHD pairings, ($\overline{r_g}$ = 0.35;

$\overline{r_e}$ = 0.26). SUD and schizophrenia are the only neuropsychiatric disorder pair where $r_e > r_g$ (0.503 - 0.332 = 0.171). Therefore, they may share parental and offspring environmental variance, some of which could be transmitted by a transgenerational effect, but do not seem to share a strong underlying genetic correlation. For example, there is a strong association between nicotine smoking and other substance use and schizophrenia.

References:

1. Visscher PM, Hill WG, Wray NR. Heritability in the genomics era — concepts and misconceptions. Nature Reviews Genetics. 2008;9(4):255-66.
2. Zhu Z, Bakshi A, Vinkhuyzen AA, Hemani G, Lee SH, Nolte IM, et al. Dominance genetic variation contributes little to the missing heritability for human complex traits. Am J Hum Genet. 2015;96(3):377-85.
3. Maki-Tanila A, Hill WG. Influence of gene interaction on complex trait variation with multilocus models. Genetics. 2014;198(1):355-67.
4. Lee SH, Ripke S, Neale BM, Faraone SV, Purcell SM, Perlis RH, et al. Genetic relationship between five psychiatric disorders estimated from genome-wide SNPs. Nature Genetics. 2013;45(9):984-+.
5. Lopez-Cortegano E, Caballero A. Inferring the Nature of Missing Heritability in Human Traits Using Data from the GWAS Catalog. Genetics. 2019;212(3):891-904.
6. Nordsletten AE, Larsson H, Crowley JJ, Almqvist C, Lichtenstein P, Mataix-Cols D. Patterns of Nonrandom Mating Within and Across 11 Major Psychiatric Disorders. JAMA Psychiatry. 2016;73(4):354-61.
7. Peyrot WJ, Robinson MR, Penninx BW, Wray NR. Exploring Boundaries for the Genetic Consequences of Assortative Mating for Psychiatric Traits. JAMA Psychiatry. 2016;73(11):1189-95.
8. Kong A, Thorleifsson G, Frigge ML, Vilhjalmsson BJ, Young AI, Thorgeirsson TE, et al. The nature of nurture: Effects of parental genotypes. Science. 2018;359(6374):424-8.
9. Purcell S. Variance Components Models for Gene–Environment Interaction in Twin Analysis. Twin Research. 2012;5(06):554-71.
10. Consortium UK, Walter K, Min JL, Huang J, Crooks L, Memari Y, et al. The UK10K project identifies rare variants in health and disease. Nature. 2015;526(7571):82-90.
11. Zuk O, Schaffner SF, Samocha K, Do R, Hechter E, Kathiresan S, et al. Searching for missing heritability: designing rare variant association studies. Proc Natl Acad Sci U S A. 2014;111(4):E455-64.
12. Wainschtein P, Jain DP, Yengo L, Zheng Z, Cupples LA, Shadyab AH, et al. 2019.

13. Cirulli ET, White S, Read RW, Elhanan G, Metcalf WJ, Tanudjaja F, et al. Genome-wide rare variant analysis for thousands of phenotypes in over 70,000 exomes from two cohorts. Nat Commun. 2020;11(1):542.
14. Zuk O, Hechter E, Sunyaev SR, Lander ES. The mystery of missing heritability: Genetic interactions create phantom heritability. Proc Natl Acad Sci U S A. 2012;109(4):1193-8.
15. Fournier T, Abou Saada O, Hou J, Peter J, Caudal E, Schacherer J. Extensive impact of low-frequency variants on the phenotypic landscape at population-scale. Elife. 2019;8.
16. Eichler EE, Flint J, Gibson G, Kong A, Leal SM, Moore JH, et al. Missing heritability and strategies for finding the underlying causes of complex disease. Nat Rev Genet. 2010;11(6):446-50.
17. Nolte IM, van der Most PJ, Alizadeh BZ, de Bakker PI, Boezen HM, Bruinenberg M, et al. Missing heritability: is the gap closing? An analysis of 32 complex traits in the Lifelines Cohort Study. Eur J Hum Genet. 2017;25(7):877-85.
18. Bloom JS, Kotenko I, Sadhu MJ, Treusch S, Albert FW, Kruglyak L. Genetic interactions contribute less than additive effects to quantitative trait variation in yeast. Nat Commun. 2015;6:8712.
19. Wolstenholme JT, Drobna Z, Henriksen AD, Goldsby JA, Stevenson R, Irvin JW, et al. Transgenerational Bisphenol A Causes Deficits in Social Recognition and Alters Postsynaptic Density Genes in Mice. Endocrinology. 2019;160(8):1854-67.
20. Wang K, Gaitsch H, Poon H, Cox NJ, Rzhetsky A. Classification of common human diseases derived from shared genetic and environmental determinants. Nat Genet. 2017;49(9):1319-25.

Supplementary Figure 1: Correlation coefficients for genetic (orange), environmental (blue) and phenotypic variance (gray) are compared between seven neuropsychiatric disorders.

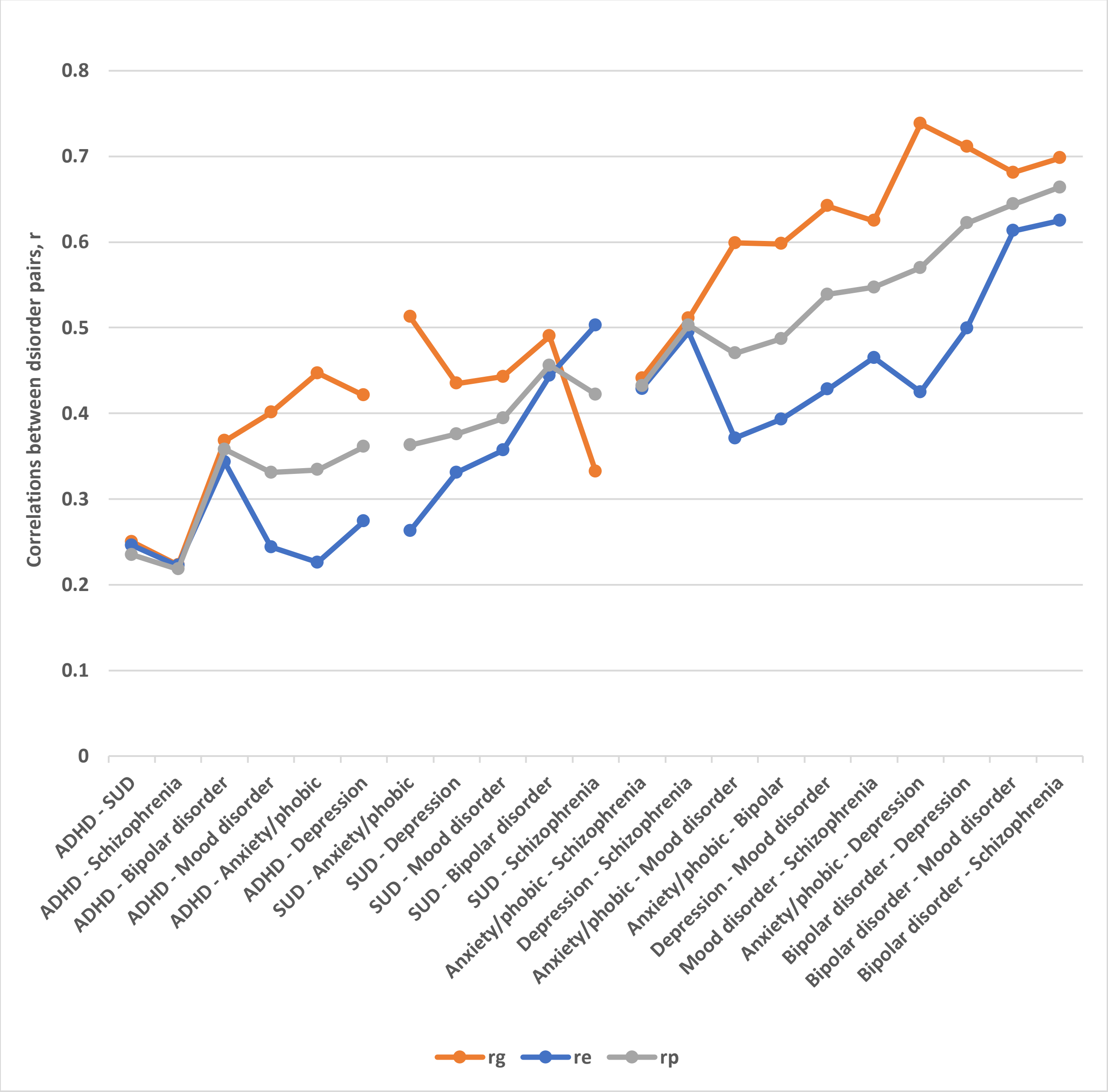

Supplementary Table 1: Effects of adding xE_p to the total genetic variance for $x = 0.25$, 0.5, 0.6 and 0.667 for 11 neuropsychiatric disorders and 3 metabolic disorders. Table shows calculated additive genetic variance G_A using estimates for heritability (H^2), shared parental environment xE_P, and total environmental variance E, in equation number [4] for 11 neuropsychiatric and 3 metabolic disorders (20).

Disorder	H^2	E_P (parents, couple)	E_S (sibling)	E_U (unique)	E (total)	G_A (0.25 E_P)	G_A (0.5 E_P)	G_A (0.6 E_P)	G_A (0.667 E_P)
ASD	0.924	0	0	0.125	0.125	0.924	0.924	0.924	0.924
ADHD	0.763	0.2	0	0.077	0.277	0.679	0.468	0.384	0.329
Eating Disorder	0.569	0.317	0	0.144	0.461	0.424	0.239	0.167	0.118
OCD	0.657	0.267	0	0.111	0.378	0.528	0.332	0.257	0.21
PTSD	0.577	0.325	0	0.128	0.453	0.426	0.35	0.156	0.104
Anxiety Phobic Disorder	0.432	0.24	0.001	0.349	0.59	0.343	0.238	0.195	0.167
Depression	0.579	0.362	0.001	0.089	0.452	0.41	0.192	0.107	0.05
Bipolar Disorder	0.676	0.269	0	0.091	0.36	0.543	0.333	0.253	0.198
Mood Disorder	0.521	0.302	0.028	0.176	0.506	0.392	0.236	0.173	0.132
Schizophrenia/Psychosis	0.562	0.253	0.025	0.189	0.467	0.454	0.308	0.251	0.212
Substance Use Disorder	0.422	0.341	0.026	0.234	0.601	0.292	0.144	0.083	0.047
Type II Diabetes (DM2)	0.561	0.283	0.112	0.074	0.469	0.437	0.276	0.212	0.171
Obesity	0.64	0.293	0	0.101	0.394	0.497	0.292	0.211	0.158
Overweight	0.576	0.329	0	0.125	0.454	0.422	0.226	0.151	0.099
